# Department of Surgery

**Published:** 1900-09

**Authors:** W. E. A. Wyman, J. A. Sloan, Robert Jay

**Affiliations:** Burlington, Wis.; San Francisco, Cal.; West Liberty, Ia.


### DEPARTMENT OF SURGERY.

By W. E. A. Wyman, M.D.V., V.S.,
BURLINGTON, WIS.

J. A. Sloan, B.Sc., M.D.V.,
SAN FRANCISCO, CAL.

Dr. Robert Jay, M.D.V.,

WEST LIBERTY, IA.

### Hoof Diseases.

Can the "practical eye" with one glance make the positive diagnosis, "hoof lameness?" This all-important question, on the whole, must be answered in the negative. The so-called practical eye is inclined to make mistakes, as the successful run of a great many diagnoses leads to an excessive confidence, which in due time means a superficial diagnostician. Thus one meets in practice a competitor's diagnosis of "navicular disease," while in reality a bit of a nail left in the hoof at the point of the frog is the explanation. It is true the horse points, has a distinct supporting leg lameness, the diseased hoof is possibly smaller than the other one, the history of former lameness on that foot, all point toward navicular arthritis. But what induces the next man to examine the foot more thoroughly? Simply because he detects excessive pulsation of the digital arteries of the lame leg, at once showing him that an active inflammation is present, navicular disease and excessive pulsation of the digital arteries being incompatible (see my *Clinical Diagnosis of Lameness in the Horse*, "Differential Diagnosis," page 74). Such cases we all have met. While a keen eye and constant practice are essential in the diagnosis of lameness, routine examinations (which mean thoroughness) are to be preferred by the vast majority of practitioners, as most of us need not hope to equal the late Professor Dick, of whom it was said that he could locate the seat of lameness by simply hearing the lame horse's footstep.

The first thing to interest the surgeon is the history of the case. The practical point here is not to ask questions beyond the sphere of the one presenting the horse for examination. A question in the wrong place may induce him to think that you are fishing, and are trying to find out too much from him. For instance, when the shoe is plainly a new one, do not ask him how long he has been shod. When pathological changes of long standing present themselves, do not ask him how long is he lame, but suggest at once that he must have been lame for a considerable period and if the owner at such a moment states that he has not been lame exactly but just limped a little, do not educate him and produce Webster's unabridged and show him the relationship of limp and lame. We are public servants, and must cater to the taste of the public—of course, without exposing one's manhood to criticism.

The essential points in the history of lameness are nine, and I quote from my *Diagnosis of Lameness in the Horse* as follows: How long is the animal lame? Under what conditions did he go lame? Was the lameness first seen while at work, or did it appear on rest? Was he shod recently? Did he sustain external violence? Did the lame leg ever show any swelling? Has the lameness decreased or increased since it first occurred? Does the lameness increase while working, or is it more pronounced after a rest? Has the animal been treated; what was the treatment?

The greatest trouble one experiences in questioning the average horse-owner lies in the fact that he is prone to give his interpretation of what are to him facts rather than actual facts which the surgeon wants. Then there is a class which expect the surgeon to be a mind-reader and who want to see how much according to their notions he knows; and finally the teamster of foreign tongue, who knows too little of anything, answering most likely any question with "Yes, sir," or "I don't know," accompanied with a broad grin. These are some of the difficulties one meets daily, at least in the practice of our large cities, while the country practitioner is more agreeably situated by reason of the entirely different relationship between himself and the owner of the animal, and while occasionally a smart farmer will cause a ripple on the otherwise smooth seas of the rural practitioner, by far most of them are only too ready to tell all they know, provided the private treatment that they instituted previous to calling in the surgeon has been followed by good results. Should their experiments have failed, then silence, and if pushed, the ancient order of prevaricators is called into action. But, after all, it is only human to hide the inferior product of one's self-recognized blunder.

Having successfully obtained the history of the case, the next thing is to locate the lame leg. To begin with, the lameness is a supporting leg lameness, increases on hard ground and (barring a few instances discussed fully elsewhere) decreases on soft ground. When travelling in a circle, with the lame leg toward the centre of the circle, supporting leg lameness increases as an excess of weight is carried at that moment by the lame leg, thus increasing intracapsular pressure. Therefore, while walking and trotting supporting leg lameness is present. At rest the animal points, as a rule, more or less permanently or intermittently, depending on the seat of the diseased area within the hoof and the intensity of the inflammation. When pivoted upon the diseased hoof sudden volar flexion, especially of the lower phalangeal articulations, shows itself—that is, the foot breaks down, as it is commonly termed. Of course, these symptoms, as well as others to be mentioned, are not alone peculiar to hoof diseases, but accompany also the diseases of the inhibitory apparatus and pathological states of the joints and bony column. Right here lies the reason why the practical eye at times may fail to land a correct diagnosis. Palpation and inspection are absolutely necessary to arrive at an up-to-date diagnosis. To the progressive man the ambiguous statement of "hoof lame" is as insufficient as the broad diagnosis of "shoulder lame." The point is to locate the exact seat of the lameness, as only then therapeutic interference and prognosis are tenable.

The next step consists of a general inspection of the animal at rest, then while walking, and, if necessary, at a trot.

What deviation from the normal as regards posture and weighing does the lame leg exhibit? Pain induces the animal to take such positions as afford him relief. As previously stated, hoof lameness means a supporting leg lameness—that is, the anterior half of the step of the lame leg is larger than the posterior half which at the same time is shortened in time. Explained by the fact that the pressure within the horny capsule, the intracapsular pressure, is greatest at the moment the lame leg is to execute its physiological function of assisting in pushing the body-weight forward and approaches the centre of gravity. To the untrained eye this appears as a retarded movement of the lame leg, and explains the rather unpardonable mistake of shoulder lameness, while in reality the animal is hoof lame—a blunder often indulged in. Should there be any doubt, have the horse moved in a circle of fair dimensions. Whenever the horse moves with the lame leg toward the centre of the circle, for instance, the left foreleg being lame, and the horse is moved toward the left, and lameness on that leg is more marked now, but when the lame leg is on the outside of the circle—that is, the horse is trotted toward the right—a supporting leg lameness is positively present. I am not now speaking of mixed lameness where both supporting leg lameness and swing-leg lameness are present, as, for instance in spavin, as here the above rule does of necessity not hold good, nor does it belong here, since hoof lameness is under discussion, the general subject of lameness having been exhaustively dealt with elsewhere.

If possible, the horse is exercised both on soft and hard ground, as a rule, hoof lameness increasing greatly on hard ground, as it induces greater concussion and intracapsular pressure, consequently increasing the pain and lameness. As the hoof-lame animal trots there is a perfect flexion of all the joints present unless other pathological states inhibit such. In the majority of hoof lamenesses a few steps suffice to bring out the lameness, while in some forms of chronic pododermatitis prolonged exercise is required to make satisfactory observations—all points to be discussed fully later on.

From an etiological and therapeutic stand-point it is well to study the motion and footing of the lame leg. Thus the toe-out and toe-in types of legs, etc., unless the bearing surface is properly levelled, assuring a balanced state, lead in due time to bruised conditions of the pododerm. Thus the diagnosis, while correct enough, is only a superficial one, as the real cause of lameness in such instances rests with an outside or inside wall too high, which, unless corrected, means a continuation of the lameness and most likely a change of veterinarians—a consummation devoutly not to be wished for, as it is much easier to get customers than to hold them.

The horse now stands still, and inspection of the lame leg, comparing it carefully with the other sound one, is the next step. Have him stand squarely on all four feet, if possible. Size up the whole of the lame leg, inspect it from in front, laterally, and from behind. Thus difference in size of muscles, atrophy, bones, and hoof is realized. Inactivity atrophy of the muscles of the shoulder, forearm, croup, and thigh, as a rule, accompany hoof lameness—sometimes of but few days' standing. Take into consideration the position of the toes and legs toward each other.

---

A rate of five dollars per week, or fifteen dollars per month, for treatment of horses is the announced offer of one of the "Lone Star State" veterinarians. The card received fails to state if this includes board.

The third edition of Duane's *Medical Dictionary* has had many valuable additions from a veterinary point of view through the assistance of Professor Leonard Pearson, who aided in its compilation.

